# Fate of long-term culture on hepatocyte-like cells generated from human adipose–derived mesenchymal stem cells

**DOI:** 10.1093/stcltm/szag061

**Published:** 2026-09-18

**Authors:** Mengling Ji, Yu Saito, Yuhei Waki, Shuhai Chen, Tetsuya Ikemoto, Takayuki Noma, Hiroki Teraoku, Shinichiro Yamada, Yuji Morine, Mitsuo Shimada

**Affiliations:** Department of Surgery, Tokushima University, Tokushima, 770-8503, Japan; Department of Surgery, Tokushima University, Tokushima, 770-8503, Japan; Department of Surgery, Tokushima University, Tokushima, 770-8503, Japan; Department of Surgery, Tokushima University, Tokushima, 770-8503, Japan; Department of Surgery, Tokushima University, Tokushima, 770-8503, Japan; Department of Surgery, Tokushima University, Tokushima, 770-8503, Japan; Department of Surgery, Tokushima University, Tokushima, 770-8503, Japan; Department of Surgery, Tokushima University, Tokushima, 770-8503, Japan; Department of Surgery, Tokushima University, Tokushima, 770-8503, Japan; Department of Surgery, Tokushima University, Tokushima, 770-8503, Japan

**Keywords:** long-term culture, adipose-derived mesenchymal stem cells, hepatocyte-like cells, bile acid

## Abstract

**Background:**

We previously established a 21-day protocol to generate hepatocyte-like cells (HLCs) from adipose-derived stem cells and are conducting preclinical studies toward their clinical application in liver failure. This study examined how long HLCs maintain viability and hepatocyte-specific functions after differentiation.

**Methods:**

Adipose-derived stem cells (2.0 × 10^6^) were differentiated into HLCs using a 3-step protocol for 21 days. After differentiation, cells were either transferred to transplantation medium (TM), used clinically, or maintained in step-3 differentiation medium (DM). Cell viability, hepatocyte-specific gene expression, and functional assays were performed on days 24 and 28. Because bile acid synthesis may induce cytotoxicity during extended culture, the expression of bile acid–related genes and transporters was evaluated on day 35.

**Results:**

Viability markedly decreased when HLCs were transferred to TM, whereas cells maintained in DM preserved viability up to day 28. The expression of mature hepatocyte markers (AAT, ALB, CPS1, and OTC) remained comparable between days 21 and 28. Hepatocyte functions, including CYP3A4 activity and ammonia metabolism, were also sustained in DM-cultured HLCs. Importantly, genes involved in bile acid synthesis and transport, which could contribute to cytotoxicity during prolonged culture, were undetectable even after 28 days.

**Conclusion:**

HLCs generated using our protocol maintained viability and hepatocyte-specific functions for at least 28 days when cultured in DM, without detectable bile acid–related gene expression. These findings suggest that transplantation timing can be flexibly adjusted for at least 1 week after the completion of differentiation.

## Graphical Abstract

**Graphical Abstract:**
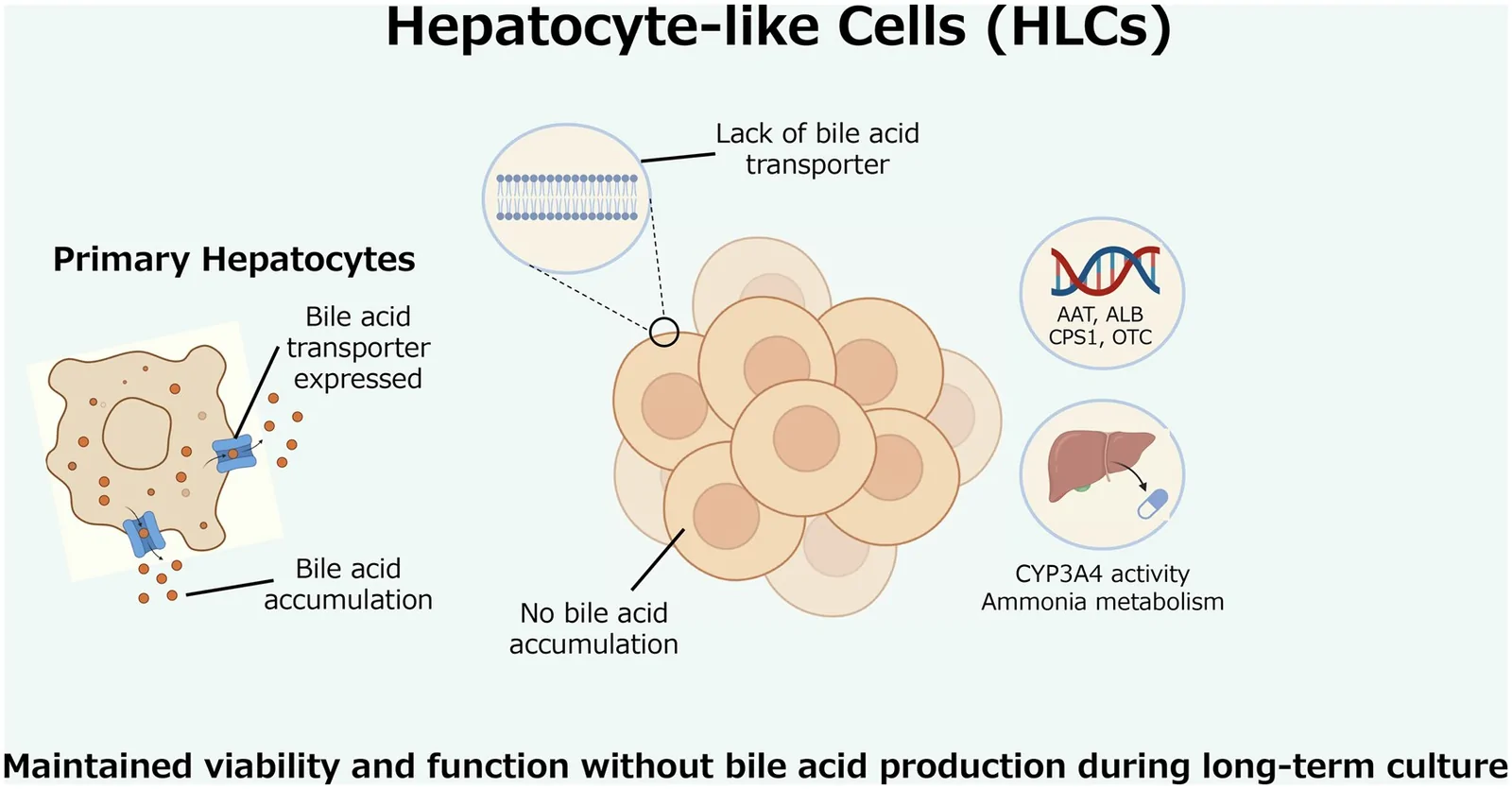

Significance statement Adipose-derived stem cells (ADSC)–derived hepatocyte-like cells (HLCs) retained hepatocyte-like characteristics for at least 1 week after completion of differentiation under the present in vitro conditions. In contrast, transfer to transplant medium rapidly impaired cell status and function. These findings help define a practical post-differentiation handling window for ADSC-derived HLCs and provide useful guidance for future translational studies.

## Introduction

Liver transplantation (LT) remains the sole definitive therapy for end-stage liver failure. In Japan, where the number of deceased donors is notably low, LT is performed almost exclusively with living donors.^1^ Although this approach is lifesaving, it carries major drawbacks, such as surgical risk to the donor, the possibility of graft rejection, the requirement for long-term immunosuppression, and significant financial costs. These challenges have intensified the demand for alternative treatment modalities that are less invasive and more broadly accessible.

In recent years, cell-based regenerative strategies using hepatocyte surrogates have gained attention as potential options to support or replace LT. Various cell types have been explored, including embryonic stem cells, induced pluripotent stem cells, and mesenchymal stem cells (MSCs). Mesenchymal stem cells are particularly promising for clinical application because they can be obtained without the need for genetic modification and pose fewer ethical and immunological issues. Among MSC sources, adipose-derived mesenchymal stem cells (ADSCs) are especially advantageous, as they can be collected through minimally invasive procedures and expanded efficiently in culture.^2–4^ We previously established a method to differentiate ADSCs into hepatocyte-like cells (HLCs). Using a defined differentiation cocktail, ADSCs can be converted into functional HLCs within 21 days without any genetic engineering.^5^ Moreover, we have shown that supplementing this cocktail with a Farnesoid X receptor agonist,^6^ or exposing cells to green light–emitting diode illumination,^7^ yields HLCs with enhanced hepatic functionality. Additionally, our work has demonstrated that constructing organoids incorporating both ADSCs and vascular endothelial cells further augments hepatocyte-related functions.^8^

When considering the clinical application of HLCs, an important clinical question arises: how long can HLCs maintain their viability and functional properties after completing the 21-day differentiation process? Whether used for autologous or allogeneic transplantation (Tx), scheduling constraints may require flexibility in determining the exact Tx date. Therefore, understanding how long HLCs can be cultured while retaining sufficient function is a critical factor for their practical use in Tx therapy. Furthermore, bile accumulation can lead to hepatocyte injury, making bile handling a key issue for HLCs Tx. Bile acids (BAs) are synthesized in hepatocytes and secreted via transporters such as bile salt export pump (BSEP) and multidrug resistance-associated protein 2 (MRP2).^9^ Because our HLCs lack cholangiocytes—which normally assist in bile metabolite processing—there is concern that prolonged culture may cause bile-related cytotoxicity.^10^ Thus, evaluating the long-term capacity of HLCs to produce and excrete bile is essential for their safe application in Tx.

In this study, we investigated how long HLCs can maintain their viability and hepatocyte-specific functions after the completion of differentiation. We also investigated whether the HLCs possess the ability to produce bile acids.

## Methods

### Ethics statements

All experiments involving human-derived samples or animals were performed in accordance with institutional, national, and international guidelines. Ethical approval for this study was obtained from the Tokushima University Hospital Ethics Committee and all associated regulatory bodies (Tokushima Clinical Trial Management System No. 3090; UMIN approval No. 000035546). All procedures adhered to the ARRIVE recommendations for animal research.

### Cell culture and differentiation of HLCs

Primary human hepatocytes (PHHs) were procured from Thermo Scientific (Waltham, MA, USA) and maintained in the supplied hepatocyte culture medium. Experiments using PHHs were carried out within 48 hours of seeding.

Human adipose-derived mesenchymal stem cells (ADSCs) were purchased from Life Technologies (Tokyo, Japan). These cells originated from adipose tissue obtained during liposuction, expanded once, cryopreserved, and later thawed according to manufacturer instructions.^11^ After recovery, ADSCs were expanded to passages 5–8 before induction. Hepatocyte-like cell (HLC) differentiation followed our established 3-step procedure. ADSCs (2 × 10^6^ cells/well) were plated onto collagen-coated 6-well plates (Nunclon Sphera Microplates) and cultured for 24 hours in serum-free DMEM/F-12.^5^ Step 1: Cells were exposed to DMEM/F-12 containing 0.5 mg/mL bovine serum albumin (BSA) (Sigma-Aldrich) and 2 µmol/L Chir99021 (Selleckchem) for 24 hours, followed by supplementation with 1% insulin–transferrin–selenium (ITS). Step 2: Cultures were switched to minimum essential medium containing BSA, ITS, osteogenic protein-2 (20 ng/mL), and FGF4 (30 ng/mL) for 5 days. Step 3: Hepatocyte maturation was induced with hepatocyte growth factor (20 ng/mL) for 5 days, followed by an additional 5 days of combined hepatocyte growth factor, oncostatin M (10 ng/mL), and dexamethasone (1 × 10^−6^ M). For extended culture experiments, 2 media formulations were used: Differentiation Medium (DM): minimum essential medium supplemented with hepatocyte growth factor, oncostatin M, and dexamethasone Transplantation Medium (TM): CMRL-1066 medium with 10% HEPES Media were refreshed every 2–3 days. Experiments using ADSC-derived HLCs were performed with 3 biological replicates derived from independent ADSC donors, and where applicable, PHH experiments were performed with 3 biological replicates derived from independent PHH donors. For each biological replicate, assays were performed in technical triplicate.

### Assessment of cell viability

Day-21 HLCs were transferred to collagen-coated 96-well plates (AGC TECHNO GLASS). Viability was assessed every 2 days starting on day 22 using the CCK-8 assay (Dojindo). After a 2-hour incubation with 10% CCK-8 reagent, absorbance at 450 nm was measured using a SoftMax Pro 7 microplate reader. Viability was calculated relative to day-21 absorbance values.

### RT-PCR analysis

Total RNA extraction was performed using the RNeasy Mini Kit (Qiagen). cDNA was synthesized with a reverse transcription kit (Applied Biosystems). TaqMan assays for AAT, ALB, CPS1, and OTC were used, with GAPDH serving as a reference gene. Relative gene expression was determined through the 2-ΔΔCt method.

### CYP3A4 activity assay

CYP3A4 enzymatic function was quantified using the P450-Glo™ Luciferin-IPA assay (Promega). Luminescence was measured with a SpectraMax i3 system and normalized to viable cell numbers.

### Ammonia metabolism assay

To evaluate ammonia detoxification, cells were incubated with 300 µmol/L ammonium chloride prepared in HBSS after 2 washes. After 24 hours, supernatants were collected and analyzed with an ammonia assay kit (Cell Biolabs). Plates without cells served as controls.

### Total bile acid quantification

Cells and media from ADSCs, HLCs, and PHHs were collected 2 days after medium replacement. Total bile acids were quantified using the Cell Biolabs Total Bile Assay Kit, with values normalized to viable cell counts.

### Immunofluorescence staining

HLCs were fixed using iPGell and 4% paraformaldehyde. Sections were incubated overnight at 4 °C with antibodies against BSEP and MRP2 (Santa Cruz Biotechnology). Alexa Fluor-conjugated secondary antibodies and DAPI were applied before imaging with a Keyence fluorescence microscope.

### Statistical analysis

Data are expressed as mean ± standard deviation (SD) of biological replicates unless otherwise stated. Statistical tests and figure generation were performed using GraphPad Prism 7 and ImageJ (v1.53). Mann–Whitney U tests were used for comparisons between 2 groups, whereas one-way ANOVA with Tukey’s post hoc test was applied to multiple-group comparisons. A *P* value <.05 was considered statistically significant. All procedures involving human-derived materials and animals were conducted in accordance with institutional and national guidelines.

## Results

### Cell viability

**Figure 1.**
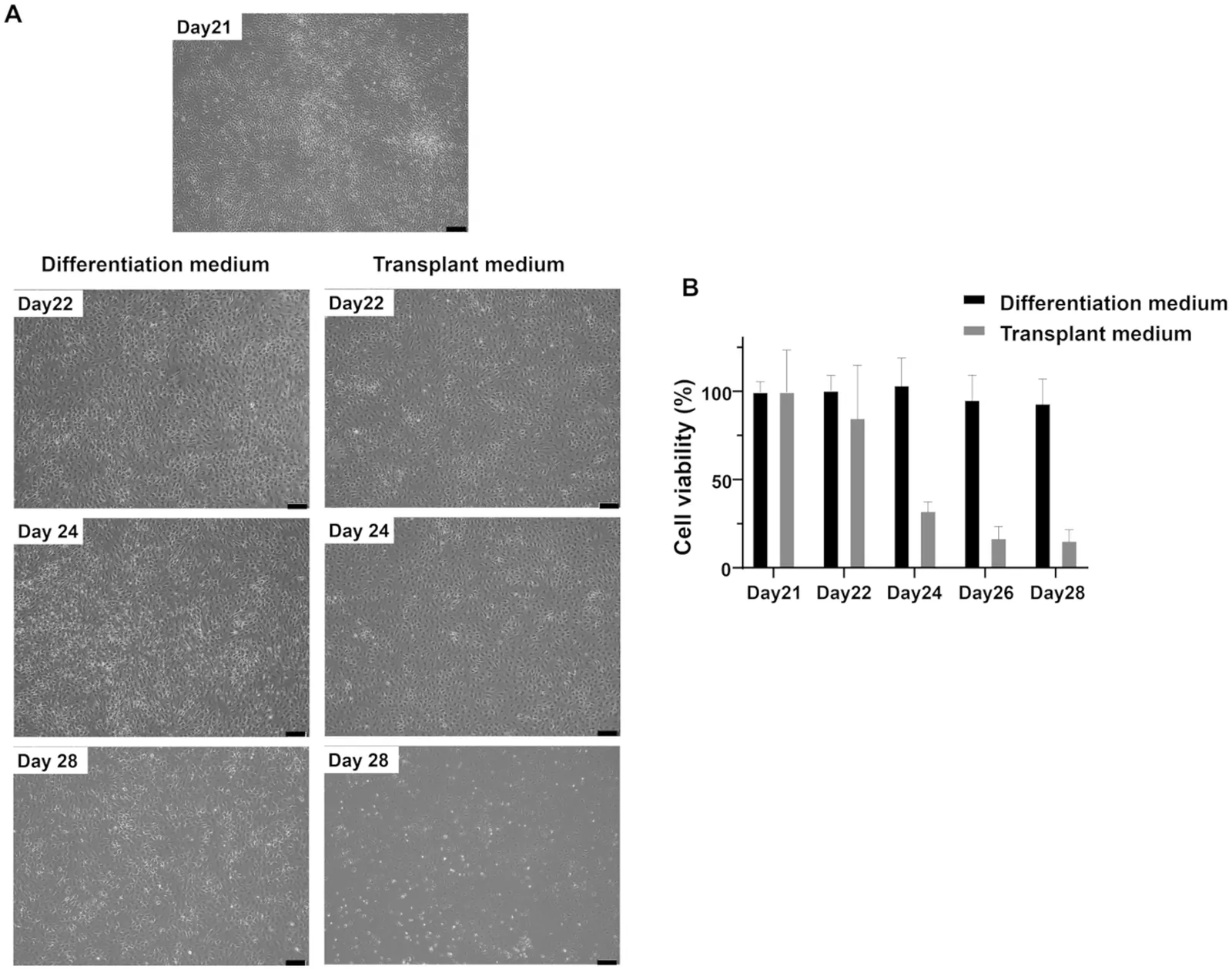
Morphology and cell viability. (A) Representative morphology of HLCs cultured in differentiation medium or transplant medium. Cells maintained stable morphology through day 28, whereas the number of viable cells progressively decreased, with more pronounced deterioration in the transplant medium group. Scale bar = 200 µm. (B) Cell viability remained stable in differentiation medium but declined rapidly in transplant medium. Data are presented as mean ± SD.

Cells cultured in DM retained normal morphology and sustained viability up to day 28. In contrast, switching the cells to TM resulted in morphological features consistent with cell death, along with a gradual, daily decline in viability (Figure 1A and B). These findings indicate that the transition to TM should be performed only immediately before transplantation, once the procedure date has been confirmed.

### Hepatocyte gene expression and function

**Figure 2.**
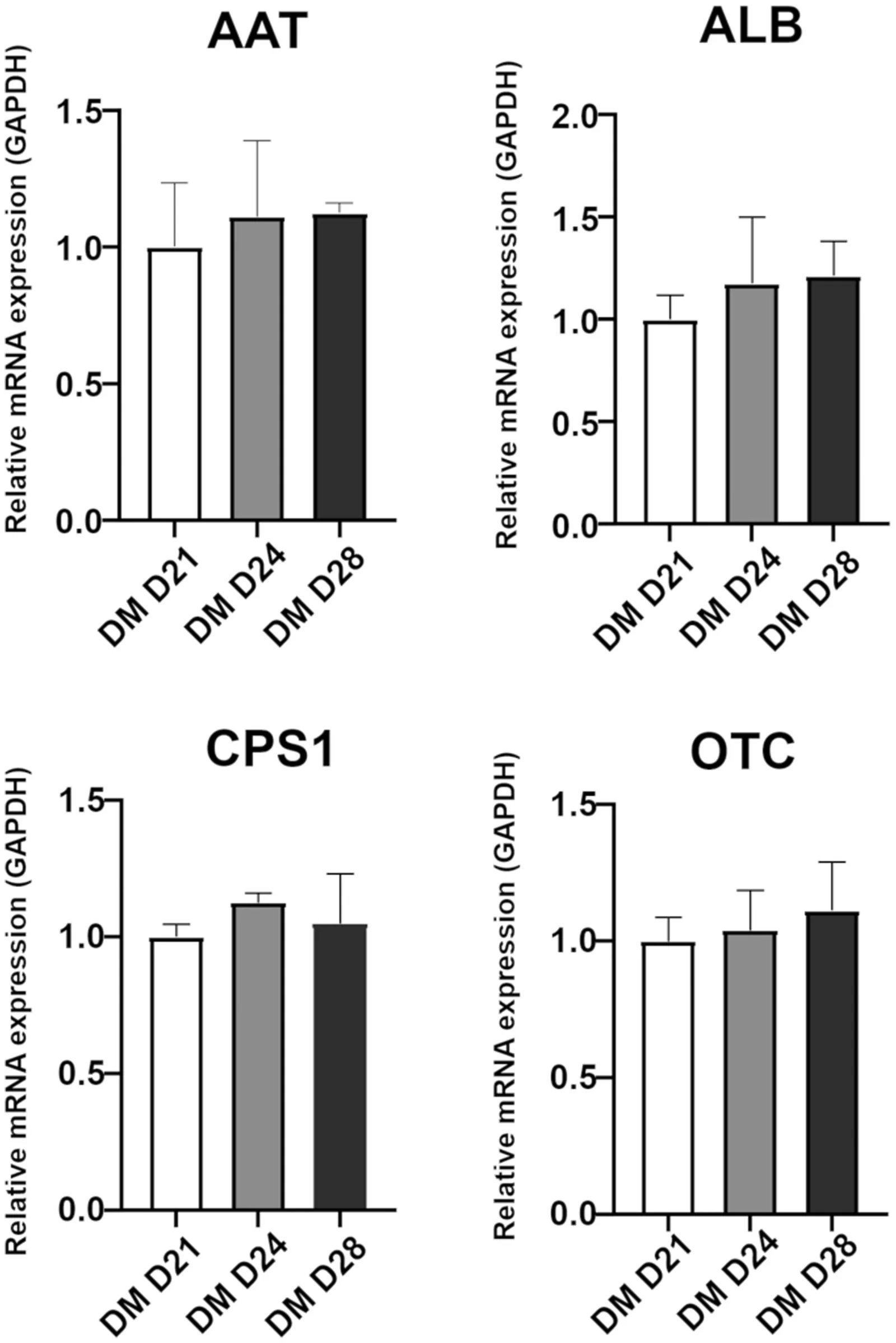
Hepatocyte maturation gene expression. mRNA expression of AAT, ALB, CPS1, and OTC in HLCs showed no significant differences between days 21 and 28 in the differentiation medium group.

**Figure 3.**
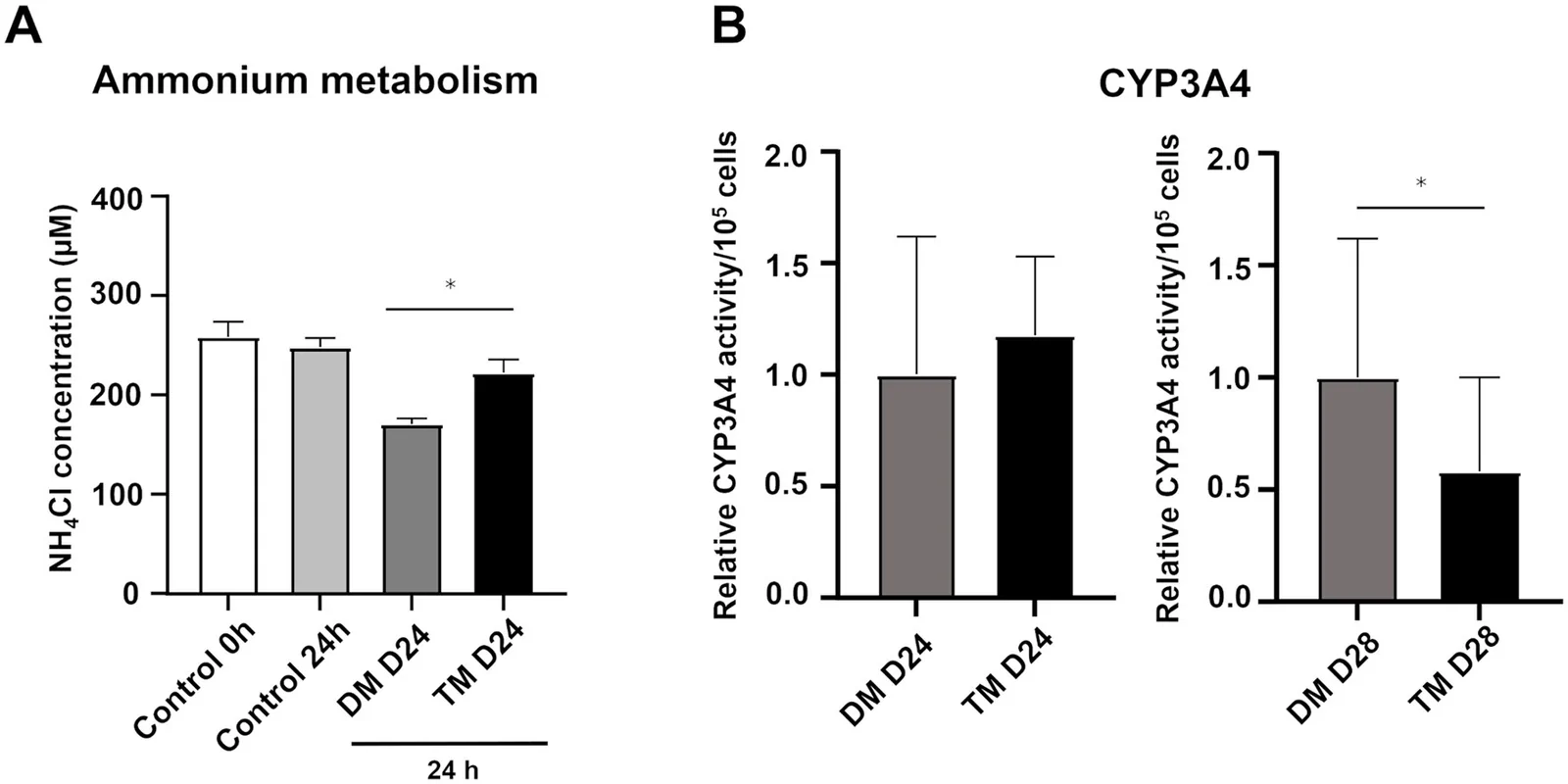
Hepatocyte functional assays. (A) Ammonia clearance was reduced in the transplant medium group compared with the differentiation medium group at day 24. (B) CYP3A4 activity was similar between groups at day 24 but declined in the transplant medium group by day 28.* *p* < 0.05.

mRNA expression levels of AAT, ALB, CPS1, and OTC in HLCs showed no significant differences between day 21 and day 28 in the DM group (Figure 2). Ammonia metabolism was reduced in the TM group compared with the DM group at day 24 (Figure 3A). CYP3A4 activity was comparable between the groups at day 24 but declined in the TM group by day 28 (Figure 3B). Collectively, these data suggest that hepatocyte-related gene expression and functional capacity in HLCs remain stable for at least 1 week when maintained in DM.

### Total bile acid concentrations in culture medium and cells

**Figure 4.**
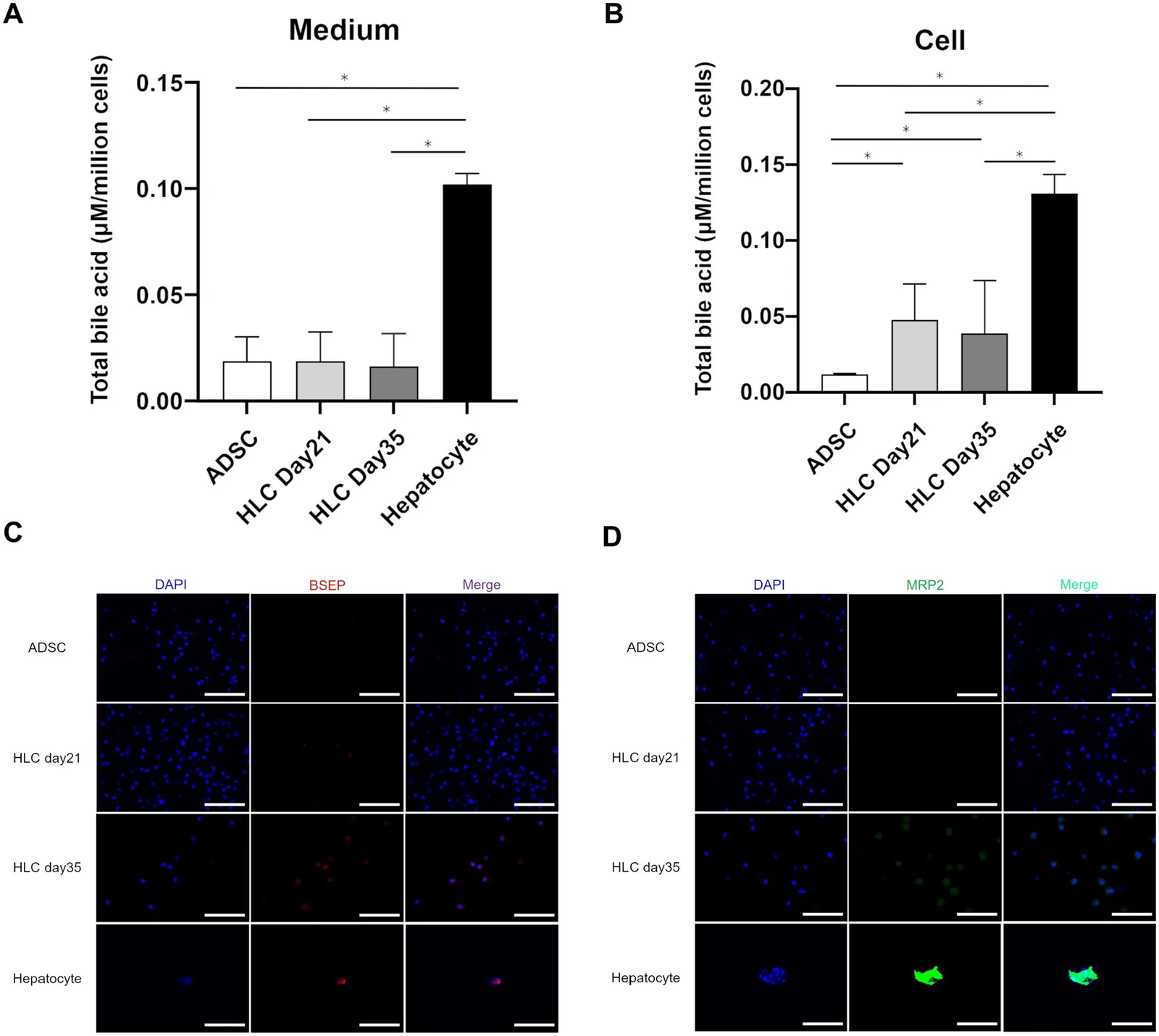
Bile acid accumulation and expression of bile acid–related transporters. Total bile acid concentrations in culture supernatants (A) and cells (B) were quantified in ADSCs and HLCs at days 21 and 35. (A) Bile acid levels in the culture supernatants did not differ significantly among ADSCs, HLCs at day 21, and HLCs at day 35. (B) Although intracellular bile acids in HLCs were slightly higher than in ADSCs, levels were markedly lower than those in PHHs. No significant differences were observed between HLCs at days 21 and 28. Immunofluorescence staining indicated bile acid–related transporters BSEP (C) and MRP2 (D). Signals were minimally detected in HLCs at days 21 and 35, in contrast to strong expression in PHHs.* *p* < 0.05.

Bile acid concentrations in the culture supernatant did not differ significantly among ADSCs, HLCs at day 21, and HLCs at day 35. Although intracellular bile acid levels in HLCs were slightly higher than those in ADSCs, they were markedly lower than those in PHHs. No significant difference was detected between HLCs at day 21 and day 28 (Figure 4A and B).

### Expression of bile acid–related transporters

Bile acid transporter expression was completely absent in HLCs, even when compared with PHHs, and no induction occurred over extended culture. These findings suggest that prolonging HLC culture to day 28 does not pose a risk of increased expression of bile acid transport–associated genes (Figure 4C and D).

## Discussion

This study demonstrates that HLCs generated through our 21-day differentiation protocol can be maintained in DM for an additional 7 days without loss of cell viability, hepatocyte maturation markers, or key hepatic functions, including ammonia metabolism and CYP3A4 activity. In contrast, once HLCs were transferred to the TM, cellular degeneration became evident within several days, resulting in a progressive decline in viability. These findings indicate that transition to TM should be performed only immediately before Tx, after the procedural schedule has been finalized. Furthermore, extended culture of HLCs did not lead to increased intracellular or extracellular bile acid accumulation, nor did it induce expression of bile acid–related transporters, suggesting that HLCs do not produce or accumulate cytotoxic levels of bile acids during the differentiation or maintenance phases.

Liver transplantation (LT) remains the only definitive therapy for end-stage liver failure; however, its clinical implementation is severely constrained by donor scarcity and procedure-related morbidity.^1^ Hepatocytes-like cells (HLCs) derived from ADSCs provide a promising alternative cell source, as ADSCs can be collected with minimal invasiveness and without ethical concerns. We previously established an induction system enabling the generation of functional HLCs without genetic manipulation.^5^ In contrast to reports indicating phenotypic instability in directly reprogrammed MSC-derived HLCs, the present study demonstrates that our HLCs maintain structural integrity and hepatic functions for at least 1 week beyond the completion of differentiation when cultured in DM. These characteristics may reduce the logistical constraints associated with cell preparation and diminish the procedural burden on recipients by eliminating the need for rapid Tx.

An important clinical consideration is whether transplanted cells produce bile acids and possess transporters necessary for their excretion. Bile congestion is a known cause of hepatocellular injury and poses a theoretical safety concern for hepatocyte Tx.^12^^,^^13^ The absence of detectable bile acid-related gene expression in ADSC-derived HLCs warrants careful interpretation in the context of hepatocyte maturation. Because mature hepatocytes normally express genes involved in bile acid synthesis and transport, the lack of detectable expression in the present HLCs may suggest incomplete maturation of bile acid handling pathways. Alternatively, this phenotype may reflect a protocol-specific feature of the current ADSC-to-HLC differentiation system. Under the present long-term culture conditions, however, this phenotype was associated with low intracellular bile acid levels and no apparent bile acid-associated cytotoxicity. Therefore, although the present HLCs may not fully recapitulate mature hepatocyte bile acid handling, limited induction of these pathways may have contributed to the preservation of morphology, viability, and hepatocyte-like function during extended culture.

This study has several limitations. First, proliferative activity of HLCs under the different post-differentiation culture conditions was not directly evaluated in the present study. Therefore, the present findings should be interpreted as reflecting maintenance of HLC morphology and function under the examined conditions, rather than as a direct assessment of proliferative status. Second, the findings are based solely on in vitro analyses, and therefore in vivo survival, engraftment, bile handling, and functional integration of HLCs remain to be determined. Further studies using animal models of hepatic metabolic disorders will be important to evaluate the in vivo survival, safety, and therapeutic efficacy of HLCs. Third, the mechanism underlying the rapid decline in HLC viability after transfer to TM was not investigated in the present study. Further studies examining apoptosis- and stress-related pathways will be needed to clarify the mechanisms contributing to decreased cell viability under TM conditions.

In conclusion, our findings indicate that HLCs can be safely maintained in DM for up to 1 week after completion of differentiation without functional deterioration, and that bile acid production remains negligible during this period. These findings support the feasibility of short-term post-differentiation maintenance of HLCs and suggest that the timing of transfer to transplant medium should be carefully considered in future translational applications.

## Conclusions

Our findings indicate that HLCs can be maintained in DM for up to 1 week after completion of differentiation while preserving hepatocyte-like characteristics under the present in vitro conditions. During this period, no detectable bile acid accumulation was observed. These findings support the feasibility of short-term post-differentiation maintenance of HLCs and suggest that the timing of transfer to transplant medium should be carefully considered in future translational applications.

## Data Availability

The data that support the findings of this study are available from the corresponding author upon reasonable request.
